# Supplementary material for: Laboratory Exploration of Several Potential Biocontrol Methods Against the Ambrosia Beetle, Euwallacea interjectus
Source: Insects. 2025 Jan 9;16(1):56. doi: 10.3390/insects16010056 (PMC11766010; doi:10.3390/insects16010056)
Supplement: Supplementary file 1 [file insects-16-00056-s001.zip › insects-3385318-supplementary.pdf]

insects

MDPI

Supplementary Material

Laboratory Exploration of Several Potential Biocontrol Methods Against the Ambrosia Beetle, *Euwallacea interjectus*

Jialin Liu [1], Nan Jiang [1], Haiming Gao [2], Shengchang Lai [1], Yang Zhou [1], Dejun Hao [1] and Lulu Dai [1,*]

1. Production of B. bassiana conidia via biphasic liquid–solid state fermentation

A small amount of *B. bassiana* was initially inoculated into SDY liquid medium (peptone 1g, dextrose 4g, yeast extract 1g, water 100mL) and cultured at 28°C in an orbital shaker at 180 rpm for 3–5 days. Commercial rice was soaked in water for 1 hour, drained, sterilized at 121 °C for 20 minutes, and cooled to room temperature under sterile conditions. The spore suspension was then added to the rice at a ratio of approximately 1:10 and mixed thoroughly. The inoculated rice was placed into disposable sterile storage bags, sealed with rubber bands, and incubated at 28 °C in the dark. Periodic kneading of the bags was performed to prevent clumping. After 2–3 days, noticeable white mycelium appeared on the rice surface. At this stage, the bags were opened to maintain aeration and reduce moisture, promoting sporulation. After approximately 7 days, a substantial amount of pale yellow or white powder, representing *B. bassiana* conidia, had formed on the rice surface. These conidia were sieved using a 200-mesh screen, freeze-dried for 48 hours, and stored at 4 °C for future use.

2. Bioassay of *B. bassiana* strain B-BB-1

Poplar log experiments: Three treatments were applied: ① Spore suspension treatment: Female *E. interjectus* adults were immersed for approximately 30 seconds in a spore suspension with a concentration of 10^8 viable spores/mL, then placed on clean paper towels to remove excess moisture. ② Spore powder treatment: A sufficient amount of spore powder was added to a 50 mL centrifuge tube. The tube was shaken thoroughly to ensure the spore powder adhered evenly to the walls. Excess spore powder was discarded. Female adults were introduced into the tube, where they acquired a coating of spore powder as they exited. ③ Control group: No treatment was applied. Logs of poplar (50 cm in height) were drilled at intervals of 5 cm along the height, with 6-8 holes per interval. Treated female beetles were placed in 0.2 mL PCR tubes with their caps on, the bottoms cut off, and the tubes inserted into the holes (Figure S1a). The top of each log was sealed with paraffin wax to minimize moisture loss. The treated logs were stood vertically in soil, maintained at room temperature, and regularly watered to keep them moist. Each poplar log was inoculated with at least 30 beetles, with at least 10 beetles per treatment, and the experiment was replicated three times with different logs. After 24 hours, boring activity was observed, and for those beetles that began boring, the PCR tube caps were opened, and a 5 mL centrifuge tube was placed around the hole to collect expelled sawdust (Figure S1b). The experimental setup is shown in Figure S2. Sawdust was collected every 2 days, dried in an oven at 75°C for 2 hours, and weighed to assess beetle activity and survival. After 10 days, the logs were dissected to determine the survival rate of the beetles and the number of offspring produced.

3. Bioassay of *S. marcescens* strain B-SM-1

To prepare the experimental setup, 60 mm PDA plates were inoculated with *F. populicola* and incubated at 28 °C for 3 to 5 days until the fungal mycelium fully covered the plates. *S. marcescens* broth serially diluted in tenfold steps to create five different concentrations, with sterile water used as a control. Using a spray bottle, each concentration (approximately 100 μL) was evenly sprayed onto the PDA plates pre-colonized by *F. populicola*. Subsequently, second- or third-instar larvae of the *E. interjectus* were uniformly introduced onto the plates, with 10 larvae per plate and three replicates for each treatment. The plates were covered and incubated at 25–28°C. Concurrently, viable counts of *S. marcescens* were conducted to confirm the exact treatment concentrations. The survival of the larvae was monitored daily, and dead larvae were promptly removed and recorded.

4. Preparation of *Serratia marcescens* broth and prodigiosin crude extract (PCE)

S. marcescens was cultured in Nutrient Broth (NB), which consists of 0.5 g beef extract, 1 g peptone, 0.5 g sodium chloride, per 100mL of water. The culture was incubated in an orbital shaker at 28 °C and 180 rpm for 2 to 3 days until the broth developed a deep red color, indicating its readiness for experimentation or for the extraction of crude prodigiosin.

The culture medium was centrifuged at 8000 rpm for 10 minutes to discard the supernatant. Approximately three times the volume of methanol was added to the pellet for extraction, followed by vortex mixing and subsequent sonication for 30 minutes. After another round of centrifugation at 8000 rpm for 10 minutes, the methanol extract was collected, and the pellet was further treated with methanol until the red color of the pellet faded, as previously described. The methanol extract was then concentrated using a rotary evaporator to remove methanol, leaving behind a concentrated solution of the PCE.

5. In vitro antagonism of F. populicola by B. bassiana and S. marcescens

B. bassiana versus F. populicola: In vitro assays were performed to assess the antagonistic interactions between *B. bassiana* and *F. populicola* on dual-inoculated plates. For each treatment, a mycelial plug of 5mm diameter was excised from the actively growing edge of a 7-day old culture on PDA using a sterile cork borer. The plug was then placed approximately 1cm from the edge of one side of a 90mm diameter Petri dish filled with PDA. For the control, PDA plates were inoculated with mycelia plugs of *B.bassiana* or *F. populicola* at one edge of each plate only. Each treatment was replicated at least five times. The plates were inverted and incubated at a constant temperature of 28 °C in the dark. Regular observations were made to monitor the appearance of inhibition zone, and the radius of the colonies were recorded to calculate the growth inhibition percentage.

S. marcescens versus F. populicola: *S. marcescens* was grown on nutrient agar (NA) for 24 hours, then streaked out on PDA with a sterile inoculation loop and the plug of the *F. populicola* was placed on the opposite side of the bacterial streak on each plate, approximately 2cm from the edge. Additionally, plates inoculated with only *F. populicola* were set as controls. Each treatment was repeated at least five times. The plates were then inverted and placed in a 28℃ constant temperature incubator for dark cultivation. Regular observations were made to check for the appearance of inhibition zone, and the radius of the colonies was recorded to calculate the growth inhibition percentage.

6. Toxicity and antifungal effects of prodigiosin crude extract (PCE)

Toxicity: As described above, several PDA plates fully colonized by *F. populicola* were prepared. The PCE was diluted with sterile water in tenfold steps to create solutions of 100%, 10%, 1%, and 0.1%, with sterile water serving as the control. Each *Fusarium*-

colonized plate received 1 mL of these solutions, which was spread evenly using a sterile spreader. Subsequently, second- or third-instar larvae of the *E. interjectus* were uniformly introduced onto the plates, with 10 larvae per plate and three replicates for each treatment. The plates were covered and incubated at 25–28°C. The survival of the larvae was monitored daily, and dead larvae were promptly removed and recorded.

Antifungal effects: Before solidification, various amounts of the PCE were added to PDA medium and mixed thoroughly to prepare PDA plates containing 0.1%, 0.2%, and 1% PCE. PDA plates without prodigiosin served as the control group, with each treatment replicated at least five times. After the plates solidified, the plug of the *F. populicola* was placed on the center of the plates. These plates were then incubated at 28 °C, and the average diameter of the fungal colonies was periodically measured to assess the antifungal efficacy.

7. Preference of two *Pyemotes* species for different stages of *E. interjectus*

In Petri dishes with a diameter of 6 cm, two female adults, pupae, and third-instar larvae of *E. interjectus* were introduced. A host with a mature opisthosoma was selected as the mite source and placed at the center of the dish. The dishes were sealed with parafilm to prevent mite escape. Each species of *Pyemotes* was replicated five times. The dishes were incubated at 28 °C. Daily observations were conducted to record the mortality rates of the three beetle stages, the number of mites attached to each stage, and the condition of the opisthosoma.

8. Life cycle of two *Pyemotes* species in parasitizing of *E. interjectus*

Several pupae of *E. interjectus* were placed in 9 cm diameter Petri dishes. A host with mature opisthosomata, actively releasing numerous offspring mites, was selected as the mite source and introduced into the dishes. Mites were released in large quantities to ensure they made thorough contact with and parasitized the pupae. After 1 day, 5 mm diameter filter paper discs were punched out and affixed to the center of sticky traps. Parasitized pupae were carefully transferred onto the filter paper discs, minimizing damage to both the pupae and the mites. Each filter paper disc contained one parasitized pupa, with 10 replicates for each *Pyemotes* species. The pupae were incubated at 25–28°C. After 15 to 20 days, when all offspring mites had fully dispersed and were captured by the sticky traps, images were taken using a Zeiss microscope. The number of mites was then counted using ImageJ.

9. Parasitism of two *Pyemotes* species on *E. interjectus*

As described above, several PDA plates fully colonized by *F. populicola* were prepared. 10 third-instar larvae of *E. interjectus* were introduced onto each plate. A host with mature opisthosomata, actively releasing numerous offspring mites, was selected as the mite source and placed in the center of the plate. Each species of *Pyemotes* was replicated at least three times, with a control group set up without mites. The plates were affixed to sticky traps to prevent mite escape. The plates were incubated at 25–28°C. Daily observations were made to record the survival of each larva, the number of mites attached to them, and the development status of the opisthosoma.

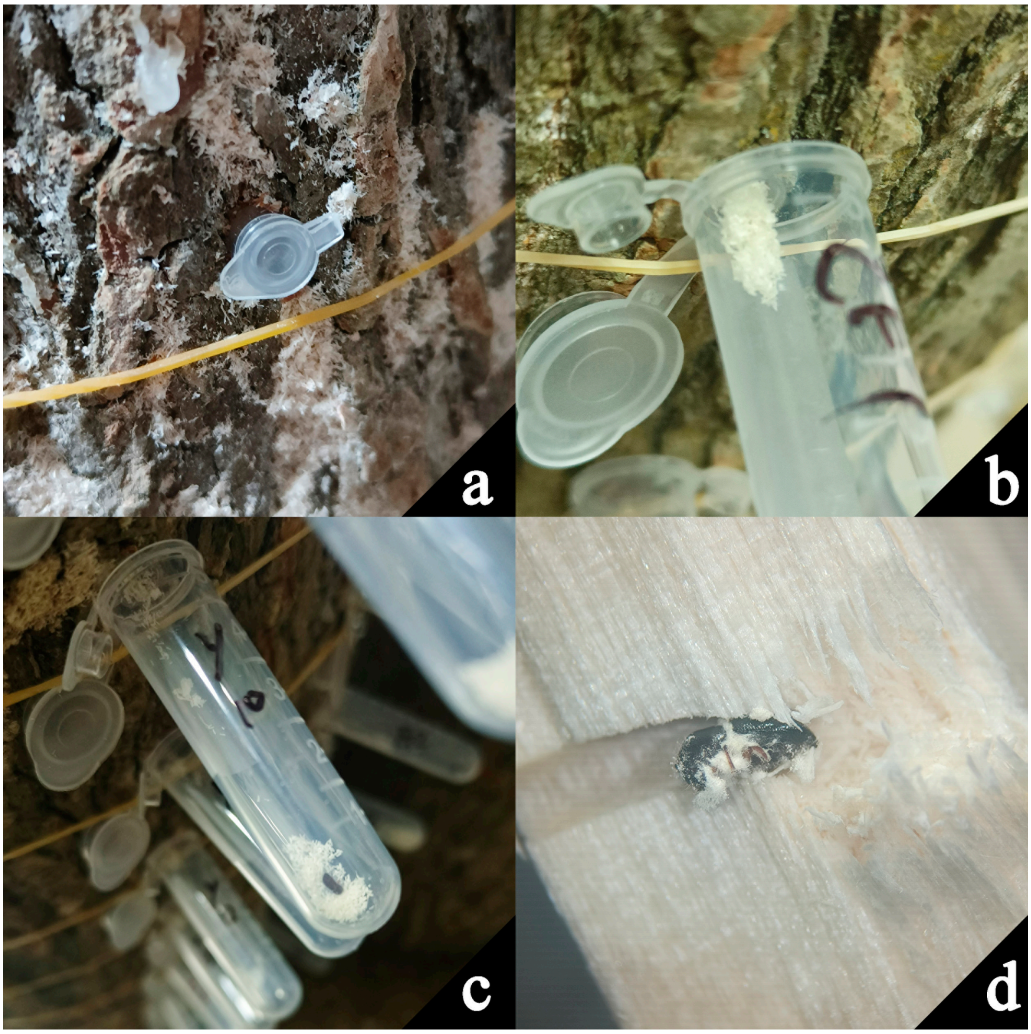

Figure S1. Operations of poplar log experiments and observations of dead beetles. (**a**) 0.2 mL PCR tube inserted into the drilled hole of the log. (**b**) 5 mL centrifuge tube used for collecting expelled sawdust. (**c**) Dead female beetle outside the gallery. (**d**) Dead female beetle at the entrance of the gallery.

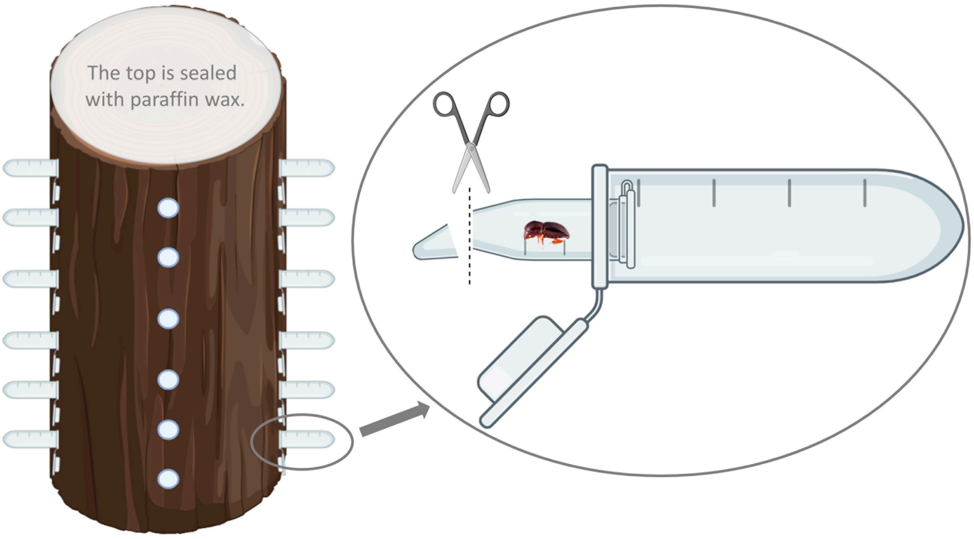

Figure S2. Diagram of the experimental setup for poplar log experiments.

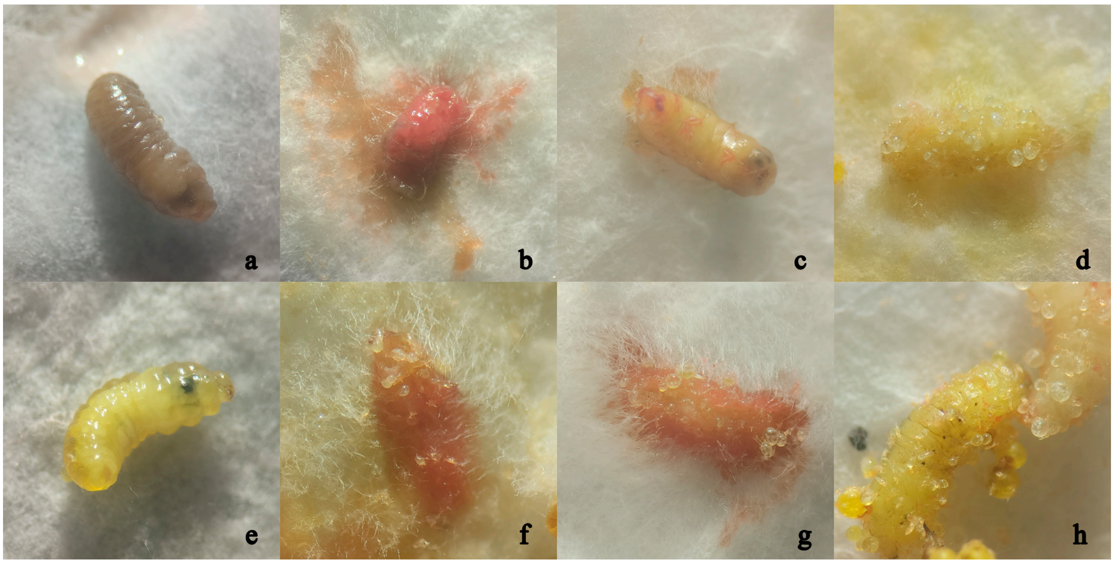

Figure S3. Microbial colonization on *E. interjectus* larva.
